# A framework for modelling whole-lung and regional transfer factor of the lung for carbon monoxide using hyperpolarised xenon-129 lung magnetic resonance imaging

**DOI:** 10.1183/23120541.00442-2024

**Published:** 2025-02-10

**Authors:** Jemima H. Pilgrim-Morris, Laurie J. Smith, Helen Marshall, Bilal A. Tahir, Guilhem J. Collier, Neil J. Stewart, Jim M. Wild

**Affiliations:** 1 POLARIS, Section of Medical Imaging and Technologies, Division of Clinical Medicine, School of Medicine and Population Health, University of Sheffield, Sheffield, UK; 2 Insigneo Institute for In Silico Medicine, University of Sheffield, Sheffield, UK

## Abstract

**Background:**

Pulmonary gas exchange is assessed by the transfer factor of the lungs (*T*_L_) for carbon monoxide (*T*_LCO_), and can also be measured with inhaled xenon-129 (^129^Xe) magnetic resonance imaging (MRI). A model has been proposed to estimate *T*_L_ from ^129^Xe MRI metrics, but this approach has not been fully validated and does not utilise the spatial information provided by three-dimensional ^129^Xe MRI.

**Methods:**

Three models for predicting *T*_L_ from ^129^Xe MRI metrics were compared: 1) a previously-published physiology-based model, 2) multivariable linear regression and 3) random forest regression. Models were trained on data from 150 patients with asthma and/or COPD. The random forest model was applied voxel-wise to ^129^Xe images to yield regional *T*_L_ maps.

**Results:**

Coefficients of the physiological model were found to differ from previously reported values. All models had good prediction accuracy with small mean absolute error (MAE): 1) 1.24±0.15 mmol·min^−1^·kPa^−1^, 2) 1.01±0.06 mmol·min^−1^·kPa^−1^, 3) 0.995±0.129 mmol·min^−1^·kPa^−1^. The random forest model performed well when applied to a validation group of post-COVID-19 patients and healthy volunteers (MAE=0.840 mmol·min^−1^·kPa^−1^), suggesting good generalisability. The feasibility of producing regional maps of predicted *T*_L_ was demonstrated and the whole-lung sum of the *T*_L_ maps agreed with measured *T*_LCO_ (MAE=1.18 mmol·min^−1^·kPa^−1^).

**Conclusion:**

The best prediction of *T*_LCO_ from ^129^Xe MRI metrics was with a random forest regression framework. Applying this model on a voxel-wise level to create parametric *T*_L_ maps provides a useful tool for regional visualisation and clinical interpretation of ^129^Xe gas exchange MRI.

## Shareable abstract

**^129^Xe MRI-based prediction of *T*_LCO_ is improved by random forest regression, and can produce regional maps of predicted *T*_LCO_, which can aid the clinical translation and comprehension of ^129^Xe gas exchange MRI** https://bit.ly/3Mu0XON

## Introduction

Pulmonary gas exchange function is usually evaluated using the carbon monoxide (CO) transfer factor (*T*_LCO_) pulmonary function test (PFT), in which the patient inhales a test gas containing 0.3% CO and 0.3% tracer gas, such as helium, and holds their breath at total lung capacity (TLC) for 10 s [1]. The dilution of the tracer gas is used to calculate the number of accessible alveolar units (*V*_A_) and the rate of disappearance of CO from the alveolar gas gives the gas exchange efficiency per unit (*K*_CO_). Together, these measurements yield *T*_LCO_, which represents the whole-lung average efficiency of gas transfer from the alveoli to the bloodstream. Although widely used [2], *T*_LCO_ measurement is a breathing test, measured at the mouth, and therefore lacks regional gas exchange information.

An alternative method to quantify pulmonary gas transfer is lung magnetic resonance imaging (MRI) with inhaled hyperpolarised ^129^Xe gas. ^129^Xe MRI is commonly used to image lung ventilation, where the presence of airway obstruction leads to poor/no ventilation in the form of low/no signal on images [3, 4]. In addition, due to its solubility in the alveolar parenchymal tissue, capillary blood plasma and red blood cells (RBCs), ^129^Xe MRI can quantitatively measure regional gas exchange limitation. ^129^Xe exhibits distinct magnetic resonance signals when present in the alveoli, when dissolved in the lung tissue and plasma (collectively referred to as “membrane” (M)) and when in the RBCs [5]. Several methods have been designed to simultaneously image xenon in each of these three environments [5–9]. The ratios of the respective signals (RBC:M, RBC:gas and M:gas) are used to quantify gas transfer and are sensitive to gas exchange limitation in COPD [10–12], interstitial lung disease [7, 13, 14] and post-COVID-19 lung disease [15–17]. However, these gas exchange ratios lack a well-defined conventional physiological interpretation, which may hinder their clinical translation.

**FIGURE 1:**
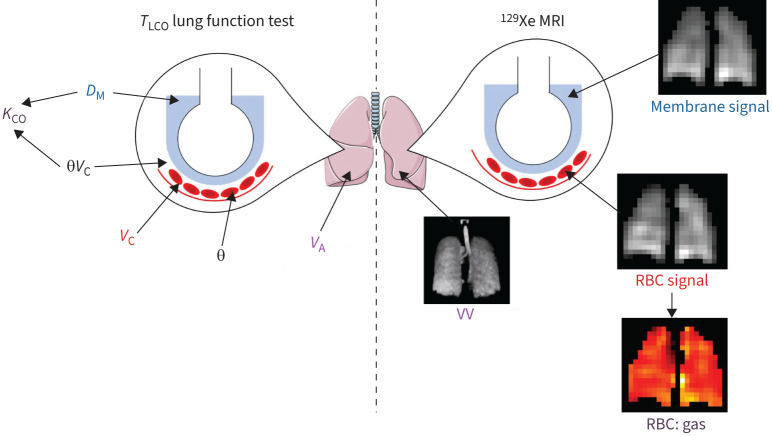
Schematic of the parallels between the underlying physiology measured by the transfer factor of the lung for carbon monoxide (*T*_LCO_) lung function test and xenon-129 (^129^Xe) magnetic resonance imaging (MRI). Like the membrane conductance (*D*_M_), the ^129^Xe membrane signal is dependent on the surface area and thickness of the alveolar membrane. The ^129^Xe red blood cell (RBC) signal is influenced by both the gas exchange across the alveolar membrane and the capillary perfusion, so can be linked to the capillary blood volume (*V*_C_). RBC:gas measures the transfer of gas from the alveolus, across the alveolar membrane and into the RBCs, so is analogous to the transfer coefficient (*K*_CO_). VV is the volume of the lung where ^129^Xe signal is detected, which is alike to the alveolar volume (*V*_A_). θ is the reaction rate of CO with the RBCs. This figure was partly generated using Servier Medical Art, provided by Servier, licensed under a Creative Commons Attribution 3.0 unported license.

There are parallels between the measurements made with ^129^Xe MRI and the constituent components of *T*_LCO_ (see Theory below), illustrated in figure 1. A model has been proposed by Wang *et al.* [18] to exploit these similarities by using features derived from ^129^Xe ventilation and gas exchange imaging, along with *V*_A_ and *K*_CO_ from PFT, to predict *T*_LCO_. However, the generalisability of this model is not clear, because the same data were used to both train the model and test its performance, leading to potentially biased results. Most commonly used *T*_LCO_ prediction models are based on participant demographics such as age and sex [19–22]. Indeed, the model-based prediction of *T*_LCO_ from ^129^Xe MRI metrics may be improved by considering age and sex, as both affect ^129^Xe MRI gas exchange metrics [23–25]. Furthermore, previous work [18] used the whole-lung average metrics from ^129^Xe imaging to predict whole-lung *T*_LCO_, which does not utilise the regional information offered by imaging. Here we propose that inputting the imaging maps to the predictive models instead would allow for regional visualisation of *T*_LCO_.

The objectives of this work were therefore to 1) evaluate the model of Wang *et al.* [18] in a large cohort of asthma and COPD patients with a rigorous testing and training group validation strategy, and 2) build upon this model to predict both whole-lung and regional *T*_LCO_ using ^129^Xe imaging and participant demographic data.

### Theory

*T*_LCO_ represents the overall conductance of CO from the alveolar gas to the pulmonary capillary blood and is made up of two components:
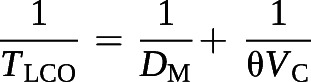
where *D*_M_ is the membrane conductance and θ*V*_C_ is the capillary blood conductance: θ is the reaction rate of CO with the RBCs and *V*_C_ is the capillary blood volume [26]. *T*_LCO_ also depends on the volume of the alveoli available for gas exchange, *V*_A_:
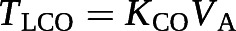
where *K*_CO_ is the transfer coefficient [1].

The model of Wang *et al.* [18] uses the following linear regression equations to predict whole-lung *T*_LCO_, where *k*_V_, *k*_M_ and *k*_R_ are coefficients found by fitting the model to measured data:
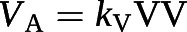

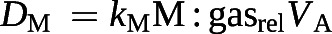

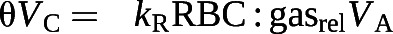
^129^Xe MRI metrics: VV is the lung ventilated volume from ventilation imaging [27] and 
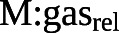
 and 
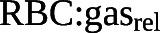
 are the dissolved ^129^Xe signal ratio values divided by a healthy reference value. Equations 4 and 5 are then substituted into equation 1:
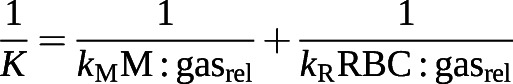
Here, the “CO” has been dropped from *K*_CO_ to specify that this is the predicted value from ^129^Xe MRI metrics. The predicted *K* and *V*_A_ values are then multiplied to give the predicted transfer factor, *T*_L_.

## Materials and methods

### Study participants

Models were trained on data from the Advanced Diagnostic Profiling (ADPro) substudy of the NOVEL observation longiTudinal studY (NOVELTY; ClinicalTrials.gov identifier: NCT02760329) of patients with asthma and/or COPD [28, 29]. Patients were recruited from primary care and had a mix of physician-assigned disease severity (mild, moderate or severe). The study involved two visits, at which participants underwent PFTs and MRI, with the second visit 12±2 months after the first. At visit one, ^129^Xe ventilation and gas exchange imaging and PFTs were performed post-bronchodilator, whereas at visit two, ^129^Xe imaging and PFTs were carried out both pre- and post-bronchodilator. Models were trained using the visit one dataset (n=165). Participants who had missing PFT or imaging data (due to missed appointments or scanner failure) were excluded and so the final training group consisted of 150 participants (table 1). 123 of 150 participants had pre-bronchodilator and 127 of 150 participants had post-bronchodilator ^129^Xe imaging and PFT data at visit two.

**TABLE 1:** Patient demographics for the testing and validation groups

	All asthma and COPD	Asthma	COPD	Asthma + COPD	Post-COVID-19 hospitalisation	Healthy
**Subjects (females), n**	150 (75)	76 (39)	24 (17)	50 (19)	23 (3)	19 (13)
**Age, years**	60.6 (21.4–82.2)	54.2±13.8	66.5±8.4	63.6±10.5	62 (42–79)	40.9±9.5
**Weight, kg**	80.7±17.4	83.7±17.0	68.4±13.5	82.0±17.5	97.0±16.0	76.8±14.5
**Height, cm**	168.5±10.4	169.0±9.8	162.0±10.4	170.8±10.3	173.4±9.1	170.5±11.3
**FEV_1_, z-score**	−0.65 (−4.49–2.66)	−0.10 (−3.84–2.66)	−1.89±1.60	−1.17±1.16	−0.70±1.00	0.13±0.75
***T*_LCO_, mmol·min^−1^·kPa^−1^**	7.56±2.55	8.48±2.18	5.16±2.46	7.01 (3.61–13.86)	6.00±1.68	8.84±2.19
***T*_LCO_, z-score**	−0.07 (−6.37–3.83)	0.29±1.14	−2.01±2.28	−0.65±1.30	−2.04±1.37	0.33±0.84
***K*_CO_, mmol·min^−1^·kPa^−1^·L^−1^**	1.38±0.32	1.53±0.24	1.10±0.35	1.27±0.28	1.30±0.17	1.52±0.16
***K*_CO_, z-score**	−0.22 (−4.83–3.00)	0.30±1.07	−1.63±1.86	−0.67±1.29	−0.52±0.78	0.09±0.68
***V*_A_, L**	5.45±1.27	5.55±1.16	3.99 (2.76–7.81)	5.72±1.22	4.58±0.87	5.82±1.33
**VV, L**	4.47±0.86	4.46 (2.08–7.28)	4.03±0.74	4.60 (2.96–7.18)	3.80±0.43	4.39±0.74
**M:gas**	0.0091 (0.0051–0.0157)	0.0099±0.0021	0.0076±0.0017	0.0090±0.0024	0.0113 (0.0089–0.0125)	0.0087±0.0012
**RBC:gas**	0.0028 (0.0012–0.0068)	0.0031 (0.0017–0.0068)	0.0017 (0.0012–0.0034)	0.0025 (0.0013–0.0058)	0.0021 (0.0013–0.0039)	0.0032±0.0006

Normally distributed variables (determined using Shapiro–Wilk tests) are presented as mean±sd, whereas non-normally distributed variables are presented as median (minimum–maximum). FEV_1_: forced expiratory volume in 1 s; *T*_LCO_: transfer factor of the lungs for carbon monoxide; *K*_CO_: transfer coefficient; *V*_A_: alveolar volume; VV: ventilated volume; M:gas: membrane to gas signal ratio; RBC:gas: red blood cell to gas signal ratio.

Model validation was carried out on a separate cohort of 42 participants. 19 of 42 participants were part of the healthy control group of the Hyperpolarised Xenon Magnetic Resonance PuLmonary Imaging in PAtIeNts with Long-COVID (EXPLAIN) study [30]. The remaining 23 participants had been hospitalised due to COVID-19 (the MURCO: MUlti-nuclear MRI in COVID-19 study [17]) and their PFT and ^129^Xe MRI data were from 1–12 months after hospital admission (median 6 months). The ADPro, EXPLAIN and MURCO studies were approved by the National Research Ethics Committee (REC reference numbers 16/EM/0439, 21/SC/0398 and 9/LO/1115).

### MRI acquisition and pulmonary function testing

Imaging was performed on a 1.5T GE HDx whole-body clinical scanner, using hyperpolarised ^129^Xe gas generated with a spin exchange optical pumping polariser [31]. ^129^Xe ventilation imaging was acquired at end-inspiratory tidal volume with a maximum dose of 0.5 L ^129^Xe and 0.5 L nitrogen (volume adjusted according to height; see supplementary table 1), using a three-dimensional (3D) steady-state free precession sequence and co-registered ^1^H anatomical imaging [32]. A multi-echo time 3D radial spectroscopic sequence [7] was used for dissolved ^129^Xe imaging, performed in the same manner with a maximum dose of 1 L ^129^Xe. For the training data, a flip angle of 40° and a repetition time of 40 ms were used for dissolved-phase imaging, whereas 22° and 15 ms were used for the validation group. More details on the differences between the two sequences are provided in the supplementary material. Additional anatomical imaging was performed using an ultrashort echo time sequence [33]. Measurement of *T*_LCO_, *K*_CO_ and *V*_A_ were performed using a Vyaire PFT Pro (Vyaire Medical, Inc., Basingstoke, UK) and in accordance with international guidelines [34].

### *T*_LCO_ prediction models

Three *T*_L_ prediction models were evaluated:

1. Physiology-based linear regression: equations 3 and 6 were fitted on the training data, first using the values of *k*_V_, *k*_M_ and *k*_R_ from [18] (model 1a) and separately using values found from a least squares solver to minimise the mean squared error (MSE) and best fit our training data (model 1b) in MATLAB (version R2022a, MathWorks, Natick, MA, USA). As in [18], RBC:gas and M:gas were normalised by healthy reference values, which were taken from a previous study [7].
2. Multivariable linear regression: features were chosen by first identifying correlated variables with a correlation matrix of possibilities (VV, M:gas, RBC:gas, age, sex, height and weight). Strongly correlated variables were removed, to avoid multi-collinearity. Separate prediction equations were then formed for *K*_CO_ and *V*_A_ by testing the predictive power of linear combinations of the remaining variables. Model fitting was performed with a linear regression solver from the scikit-learn Python toolbox (Python 3.9.12) [35].
3. Random forest regression: this is an ensemble machine-learning algorithm which combines predictions from many uncorrelated decision trees to output a prediction or classification [36]. Two regression models were trained using scikit-learn [35], to predict *K* and *V*_A_ separately, using the features identified from linear regression modelling. Tree splitting was based on minimisation of the MSE and model hyperparameters (supplementary table 2) were tuned using a grid search.

For both models 2 and 3, RBC:gas and M:gas were not normalised by the healthy reference values.

### Model training and validation

The three models were initially trained using five-fold cross-validation: the training data were split randomly into five folds of 30 participants (stratified such that each group contained approximately the same proportion of asthma, COPD and combined asthma and COPD patients), and the models were fitted on four of the folds, with the remaining fold used to test the fit performance. This was repeated five times so that each fold acted as the testing data once. The model with the lowest MSE across the cross-validation folds was chosen as the final model and this was subsequently retrained on the entire training data and evaluated by applying to the validation set and ADPro visit two data.

### Regional *T*_LCO_ mapping

**FIGURE 2:**
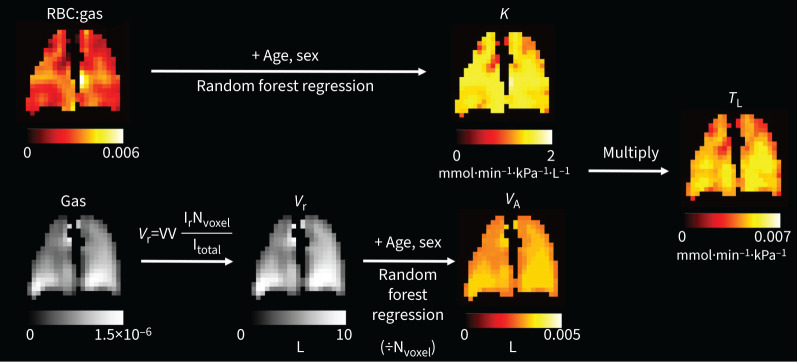
Regional random forest model: information on regional red blood cell (RBC) uptake and gas signal distribution from dissolved xenon-129 (^129^Xe) imaging was utilised to produce regional maps of transfer factor (*T*_L_). The transfer coefficient (*K*) prediction pipeline was applied to every voxel of the RBC:gas map, along with patient age and sex, to output a map of predicted *K*. For the prediction of accessible alveolar volume, an extra step (equation 7) was required to convert the gas signal map into a map of ventilation distribution (*V*_r_), which had the required units of litres and order of magnitude. This involved finding the signal intensity of each pixel (I_r_), dividing this by the mean signal intensity (I_total_/N_voxel_) and multiplying this fraction by the ventilated volume (VV) from ^129^Xe ventilation imaging. The *V*_A_ random forest prediction was then applied to each voxel of this map, along with patient age and sex. The resulting map was renormalised by N_voxel_ so that it represented the ventilation per voxel and summed to give predicted *V*_A._ This was then multiplied with the *K* map to obtain a map of *T*_L_.

To derive regional maps of *T*_LCO_, the final trained *K*_CO_ and *V*_A_ models were applied to each voxel of the RBC:gas map and a “relative” ventilation map determined from the gas-phase images of the gas exchange acquisition (figure 2). The maps were masked by applying a noise threshold to the M signal images. Ventilation distribution maps were found using equation 7:
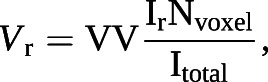
where *V*_r_ is the relative ventilation at position r = (x, y, z), I_r_ is the gas signal intensity at position r, N_voxel_ is the total number of ventilated voxels (*i.e.* voxels in the lung mask) and I_total_ is the total gas signal intensity. This intermediate step was required so that the input to the *V*_A_ model (*V*_r_) had the same units and order of magnitude as the VV data that the model was trained on. So that the *V*_A,r_ maps had a clearer physiological meaning (VV per voxel), the initial *V*_A_ model output was scaled by N_voxel_. The whole-lung predictions can be recovered from the regional maps by summing the fractional *T*_L_ and *V*_A_ values (*T*_L__r_ and *V*_A,r_) and averaging the rate *K* (*K*_r_):
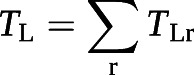

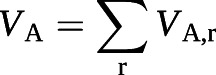

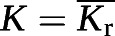


### Statistical analysis

The predictive power of the models was assessed by calculating the MSE and R^2^ of the linear fit of predicted and measured *T*_LCO_, the mean absolute error (MAE) between the predicted and measured values and their Bland–Altman bias. Statistical analysis was carried out with RStudio (R version 4.3.0).

## Results

### Validation of physiological model

Model 1a: the model using linear regression coefficients from Wang *et al.* [18] (*k*_V_=1.47, *k*_M_=3.55 mmol·min^−1^·kPa^−1^·L^−1^ and *k*_R_=4.55 mmol·min^−1^·kPa^−1^·L^−1^) did not fit our training data well (table 2; MAE=2.66 mmol·min^−1^·kPa^−1^). Model 1b: the prediction accuracy was improved by refitting the values of *k*_V_, *k*_M_ and *k*_R_ on our dataset (MAE=1.24±0.15 mmol·min^−1^·kPa^−1^); the refitted values were: *k*_V_=1.21±0.01, *k*_M_=4.51±0.26 mmol·min^−1^·kPa^−1^·L^−1^ and *k*_R_=2.97±0.13 mmol·min^−1^·kPa^−1^·L^−1^. Linear regression and Bland–Altman analysis of the measured and predicted *T*_LCO_ values are shown in supplementary figure 1.

**TABLE 2:** Evaluation of the four transfer factor prediction models on the training data

Model	MSE (mmol^2^·min^−2^·kPa^−2^)	R^2^	MAE (mmol·min^−1^·kPa^−1^)	Bias (LOA) (mmol·min^−1^·kPa^−1^)
**1a**	3.49	0.470	2.66	2.41 (−1.56–6.39)
**1b**	1.63±0.31	0.604±0.028	1.24±0.15	−0.14 (−3.25–2.97)
**2**	1.16±0.23	0.739±0.069	1.01±0.06	−0.02 (−2.55–2.52)
**3**	1.13±0.24	0.744±0.063	0.995±0.129	0.02 (−2.50–2.54)

Model 1a: physiological model with coefficients from Wang *et al.* [18]; 1b: physiological model with refitted coefficients; 2: multivariable linear regression; 3: random forest regression. For models 1b, 2 and 3, the mean and sd across the five cross-validation folds is given. MSE: mean squared error; R^2^: coefficient of determination; MAE: mean absolute error; LOA: limits of agreement.

### Model development and validation

Model 2: M:gas and height were not included as features in the multivariable linear regression model because of their dependence on other variables. More information on the choice of model features is provided in the supplementary material. The final predictive equations were:
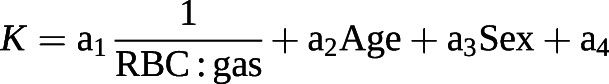
and
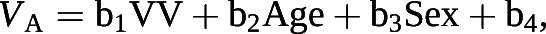
where sex is 0 for males and 1 for females. The fitted values of a_1,2,3,4_ and b_1,2,3,4_ can be found in supplementary table 3. Including participant age and sex as predictors improved the prediction of *T*_LCO_ compared with model 1b (MAE=1.01±0.06 mmol·min^−1^·kPa^−1^).

**FIGURE 3:**
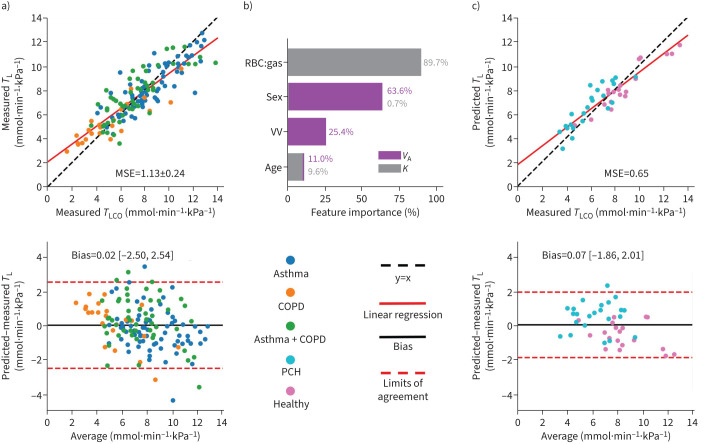
Random forest regression model. a) Linear regression (top) and Bland–Altman (bottom) plots of the measured and random forest-predicted transfer factor values for the training data. b) ranked importance of the prediction variables. c) Linear regression (top) and Bland–Altman (bottom) plots for the measured and random forest-predicted transfer factor values for the validation group. *T*_L_: transfer factor of the lungs; MSE: mean squared error; *T*_LCO_: *T*_L_ for carbon monoxide; RBC: red blood cells; *V*_A_: alveolar ventilation; V: ventilated volume; PCH: post-COVID-19 hospitalisation; *K*: transfer coefficient.

Model 3: the random forest regression model performed slightly better than linear regression (MAE=0.995±0.129 mmol·min^−1^·kPa^−1^; figure 3a) so was chosen as the final model. The most significant predictor of *K* was RBC:gas, whereas sex was the highest-ranking predictor of *V*_A_ (figure 3b). This is expected because sex is strongly correlated with height, which in turn is a significant predictor of lung volume [37]. The model also performed well when applied to the validation data (figure 3c; MAE=0.840 mmol·min^−1^·kPa^−1^, MSE=0.647·mmol^2^·min^−2^·kPa^−2^, R^2^=0.828). For completeness, models 1a, 1b and 2 were also evaluated on the validation data (supplementary figure 1) and the performance of model 2 was found to be slightly better that of model 3: MAE=0.744 mmol·min^−1^·kPa^−1^, MSE=0.452·mmol^2^·min^−2^·kPa^−2^, R^2^=0.877.

### Regional *T*_LCO_ prediction

**FIGURE 4:**
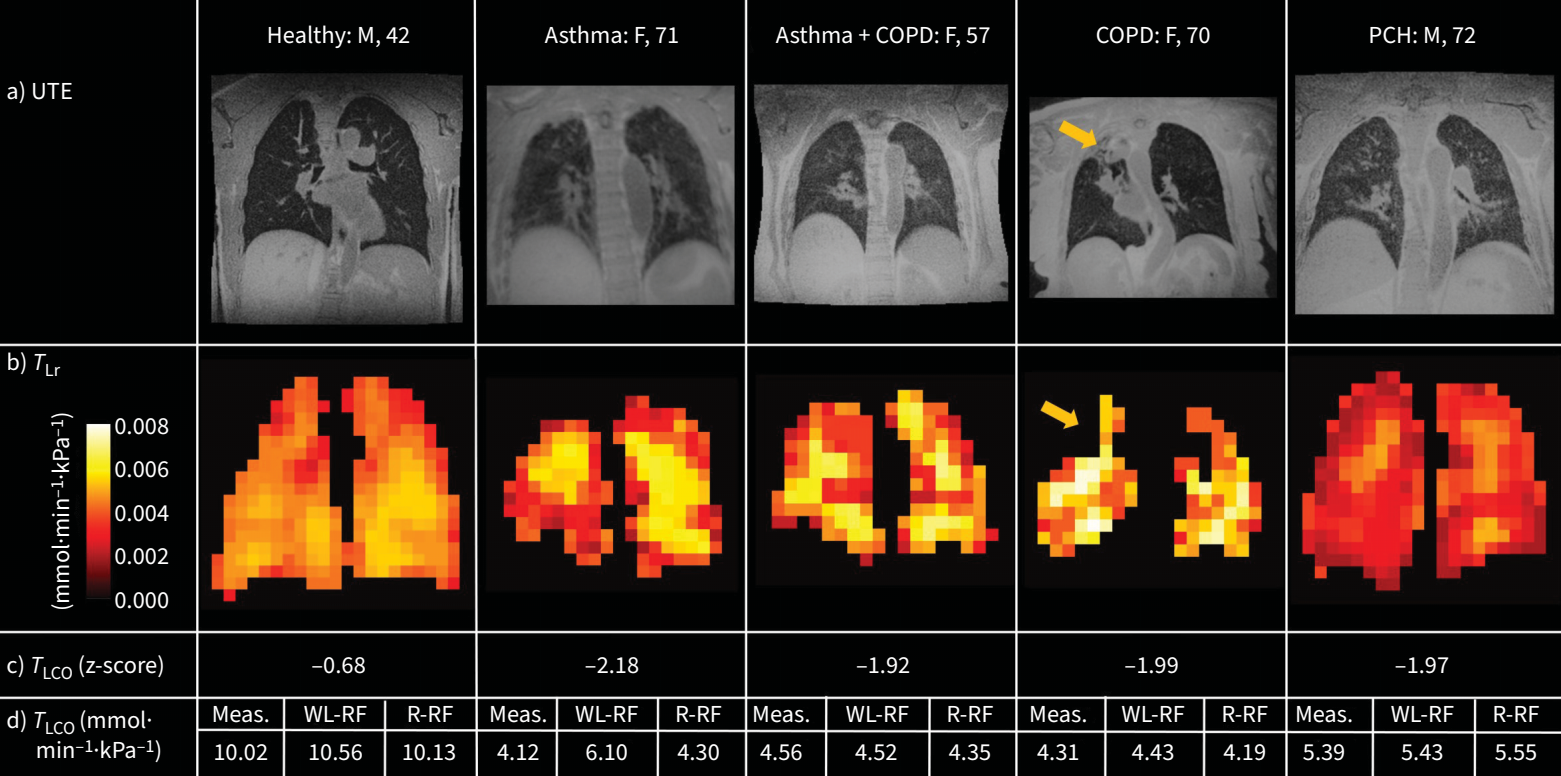
a) Ultrashort echo time (UTE) lung structure images and b) random forest regression-predicted transfer factor of the lung (*T*_L_) maps for five participants and their diagnosis, sex and age, and c) *T*_L_ for carbon monoxide (*T*_LCO_) z-score at visit one. The fourth patient shown in a) had a lack of xenon-129 signal in the upper right lung due to underlying structural changes (yellow arrows). d) The measured and estimated *T*_L_ values for each participant are given, where “WL-RF” refers to the value from the whole-lung random forest model and “R-RF” refers to the value from the sum of *T*_L__r_ from the regional random forest model over all voxels. M: male; F: female; PCH: post-COVID-19 hospitalisation.

**FIGURE 5:**
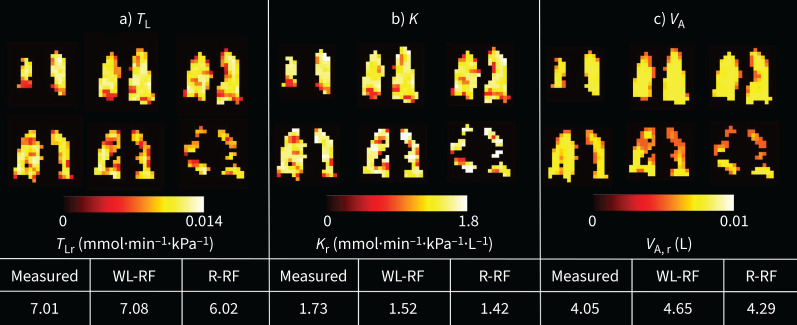
Random forest regression modelled a) transfer factor of the lung (*T*_L_), b) transfer coefficient (*K*) and c) alveolar volume (*V*_A_) maps for a 57-year-old female patient with both asthma and COPD for six lung slices, plus the modelled and measured whole-lung values at visit one. “WL-RF” refers to the value from the whole-lung random forest model and “R-RF” refers to the value from the regional random forest model. Although this patient had a normal *T*_L_ for carbon monoxide (*T*_LCO_) (as measured by pulmonary function testing), their *T*_L_ map indicates reduced gas exchange. The *K* map shows a heterogeneous gas transfer rate which does not match the ventilation distribution.

Example predicted *T*_L_ maps demonstrating differences between four participants with different pathology, but similar *T*_LCO_ z-scores, and one healthy volunteer are shown in figure 4. With the addition of regional gas exchange information, the performance of the random forest model was not improved when compared with the whole-lung model, but was still reasonably good: MAE=1.18 mmol·min^−1^·kPa^−1^, MSE=1.26·mmol^2^·min^−2^·kPa^−2^, R^2^=0.736. It also performed well when applied to visit two data from the same study, for both pre-bronchodilator (MAE=1.30 mmol·min^−1^·kPa^−1^, MSE=1.17 mmol^2^·min^−2^·kPa^−2^, R^2^=0.747) and post-bronchodilator (MAE=1.14 mmol·min^−1^·kPa^−1^, MSE=1.13 mmol^2^·min^−2^·kPa^−2^, R^2^=0.756) data. Linear regression and Bland–Altman plots comparing the measured, whole-lung random forest-predicted and regional random forest-predicted *T*_L_ values for both visits are shown in supplementary figure 2. Example *T*_L_-constituent *K*_CO_ and *V*_A_ maps for one participant with both asthma and COPD are shown in figure 5. This person had normal measured *T*_LCO_ (z-score=0.48), but their maps reveal regional heterogeneity in both ventilation and gas transfer.

**FIGURE 6:**
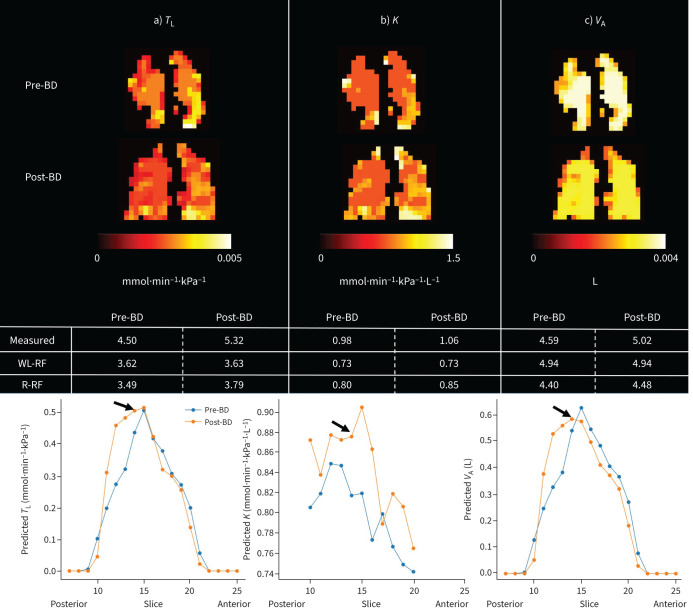
Predicted maps of a) transfer factor of the lung (*T*_L_), b) transfer coefficient (*K*) and c) alveolar volume (*V*_A_) for a single lung slice for a 61-year-old female with both asthma and COPD pre- and post-bronchodilator (BD). The sum (mean for *K*_r_) for each slice (posterior to anterior) is plotted, showing the change in distribution following BD administration. The lung slice is indicated with an arrow. These plots show that *V*_A_,_r_ and *T*_L__r_ increase in the posterior lung following BD, while slightly decreasing in the central and anterior slices.

Figure 6 shows the predicted *T*_L_, *K* and *V*_A_ maps for a single slice for a participant with both asthma and COPD before and after bronchodilator administration. The maps show an increase in the lung mask area post-bronchodilator, but the gas signal intensity per voxel decreases because the same amount of gas is distributed over more voxels due to the opening of previously unventilated airways. Changes in the regional distribution of *T*_L_, *K* and *V*_A_ following bronchodilator can be assessed by considering the predicted values for each lung slice (figure 6, bottom panel).

## Discussion

In this work, we have demonstrated that random forest regression improves prediction of *T*_L_ from ^129^Xe MRI metrics when compared with a physiology-based model. We then go on to apply this model to create maps of *T*_L_, *K* and *V*_A_. This approach provides a valuable means to visualise these clinical lung physiology metrics at a regional level, allowing the contributions from the ventilation distribution and gas uptake rate to be examined, and links measures from ^129^Xe gas exchange imaging to well-established physiological quantities.

Three models for predicting *T*_L_ from ^129^Xe MRI data were evaluated. The coefficients from the previously proposed model [18] did not fit our data well, likely due to differences in MRI acquisition strategies/parameters and participant disease aetiologies; the previous model was trained on data from healthy participants and those with obstructive, restrictive and pulmonary–vascular lung disease. It is also possible that, by not using cross-validation, the coefficients in [18] were reached by finding a suboptimal local minimum of MSE. In contrast to [18], we found *k*_M_>*k*_R_, which suggests a greater contribution to the total impedance from the membrane than the capillary blood, or ventilation–perfusion mismatch.

The addition of participant age and sex in the multivariable linear and random forest regression models further improved prediction accuracy when compared with the physiology-based model. Age appeared in both the *K* and *V*_A_ prediction, suggesting that *T*_LCO_ has a second-order age dependence. An age^2^ dependence represents an accelerated loss of lung function with age and is also found in the *T*_LCO_ prediction equations of Munkholm *et al.* [22]. All models had small sd across the five cross-validation folds, demonstrating good accuracy and minimal overfitting, but the best-performing model for the training data was random forest regression. This may be because the model is able to account for nonlinear relationships [36]; whereas equations 3 and 12 assume a linear relationship between VV (acquired at functional residual capacity +1 L) and *V*_A_ (acquired at TLC), the lung volume dependent pathophysiology in obstructive lung disease means that the relationship is likely to be more complicated.

Beyond improved modelling, a further novel aspect of this work is the application of the random forest prediction model to produce spatially localised *T*_L_, *K* and *V*_A_ parametric maps, allowing for regional visualisation of otherwise global metrics. Combining the information from dissolved-phase and ventilation ^129^Xe MRI, along with patient age and sex, into a parametric map with defined units may provide a way for respiratory physicians to easily interpret ^129^Xe gas exchange MRI. *T*_L_ mapping could assist in the phenotyping of patients and in assessing longitudinal changes and treatment response, especially for patients with both gas exchange limitation and ventilation heterogeneity. One of the key assumptions in the *T*_LCO_ PFT is that the inspired test gas is homogeneously distributed throughout the lung. However, in obstructive lung disease, ventilation is heterogeneous and so this assumption may not be appropriate and can lead to an underestimation of *T*_LCO_ [38]. Contrarily, our models use regional gas exchange measurements from dissolved ^129^Xe MRI to estimate transfer factor of the lung *T*_L_, which are intrinsically sensitive to the distribution of ^129^Xe gas in the lungs. This may explain why the models consistently overestimated *T*_L_ for COPD patients with low measured *T*_LCO_ values.

*T*_L_ was often underestimated for participants with high measured *T*_LCO_. This may be due to the larger error on predicted *T*_L_ 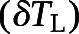 for higher *T*_LCO_ values. *T*_L_ was found from the product of the predicted *V*_A_ and *K* values/maps and so the error on these components is propagated through to *T*_L_ according to error propagation formula:
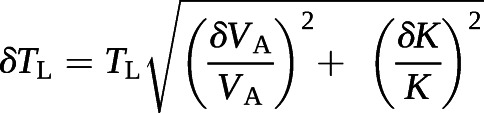
Inherent differences between ^129^Xe MRI and *T*_LCO_ measurement, such as the diffusivity and solubility of the gases used and the lung volumes and body positions, may also limit the predictive power of the models. Although the random forest and linear regression models improved the prediction of *T*_L_, with these models the ability to estimate the membrane and capillary blood conductance is lost. Another limitation of our work is the requirement for both ventilation and gas exchange ^129^Xe imaging, which necessitates two separate acquisitions with up to 1.5 L of xenon. At present it is not practical to calculate VV from the gas exchange imaging gas-phase image, because the image resolution (1.25 cm^3^ voxel size) is too low for reliable registration with the anatomical proton image. However, image acceleration techniques such as compressed sensing and rapid spiral k-space encoding gradients may make a combined ventilation and gas exchange imaging sequence feasible [39].

The models in this work were trained solely on patients with obstructive lung disease and their application to patients with restrictive and pulmonary–vascular disease and patients from a different site has not been explored. There are still considerable differences between the ^129^Xe MRI sequences and acquisition parameters used between sites, which may limit the application of our models to external data; however, efforts towards harmonisation are being made through the ^129^Xe MRI Clinical Trials Consortium [40]. Hence, it is possible that some retraining could be required to tune the model parameters for external data, but we anticipate that the models themselves and the principle of using regression modelling to map *T*_L_ should be generalisable. We have attempted to reduce overfitting by using a five-fold cross-validation training strategy and used training data from a heterogeneous population with a large range of *T*_LCO_ values (1.60–13.86 mmol·min^−1^·kPa^−1^). The models performed well on validation data from two different clinical studies, with different patient groups and acquisition parameters to the training data, suggesting generalisability to other lung diseases. This is despite the bias between the datasets from differences in repetition time and flip angle in the dissolved ^129^Xe imaging sequence. Testing the models on different patient cohorts and on external data is required to fully assess their generalisability.

In conclusion, regional *T*_LCO_ can be modelled from ^129^Xe MRI metrics for patients with obstructive lung disease. Prediction accuracy was greatest with a random forest regression model which used patient age, sex and ^129^Xe MRI-derived VV and RBC:gas as prediction variables. The ability of this model to generate *T*_L_ maps presents a useful tool for visualisation and interpretation of regional *T*_L_ limitation and should help facilitate the clinical translation of ^129^Xe gas exchange MRI.

## Supplementary material

10.1183/23120541.00442-2024.Supp1 **Please note:** supplementary material is not edited by the Editorial Office, and is uploaded as it has been supplied by the author.
Supplementary material 00442-2024.SUPPLEMENT
