# Supplementary material for: A framework for modelling whole-lung and regional transfer factor of the lung for carbon monoxide using hyperpolarised xenon-129 lung magnetic resonance imaging
Source: ERJ Open Res. 2025 Feb 10;11(1):00442-2024. doi: 10.1183/23120541.00442-2024 (PMC11808933; doi:10.1183/23120541.00442-2024)
Supplement: Supplementary file 1 [file 00442-2024.SUPPLEMENT.pdf]

# Supplementary Material for 'A framework for modelling whole-lung and regional $TL_{CO}$ using hyperpolarised $^{129}$Xe lung MRI', J.H. Pilgrim-Morris et al

## Methods

MRI Acquisition for Training and Validation Data:

The training data were acquired with a 3D four-echo spectroscopic imaging sequence with TR = 40 ms and a flip angle of 40$^{o}$ centred on the dissolved $^{129}$Xe resonance [1]. The validation data were acquired with an updated version of this sequence [2]. The changes made in the updated sequence are summarised below:

1. A TR of 15 ms and a flip angle of 22$^{o}$ were used.
2. The amplitude-modulated composite RF excitation pulse was replaced with a frequency-tailored RF pulse with 1% excitation of the gas-phase was used, which removed the need for interleaved gas and dissolved-phase excitation.
3. The number of radial projections was increased from 332 to 934.
4. Calibration spectra were integrated into the start of the imaging sequence rather than being acquired separately.

The different TR and flip angle used mean that there is likely to be some bias between the datasets, because the combination of these two parameters determines where on the dissolved $^{129}$Xe uptake curve the measurement samples [3]. The average difference in mean RBC:Gas between the two sequences was evaluated in 6 healthy volunteers and was found to be ~15%. This variability is similar to the repeatability of RBC:Gas and does not seem to have limited the applicability of our models to the validation data.

Feature Choice:

To choose the features (variables) for the linear regression model, a Spearman's correlation matrix was calculated for all possibilities (RBC:Gas, M:Gas, VV, age, sex and height). RBC:Gas and M:Gas were strongly correlated (Spearman's r = 0.57) and so were age and height (r = -0.75) and VV and height (r = 0.62). To avoid multicollinearity problems, the model features should not be strongly interdependent.

RBC:Gas was chosen to be included over M:Gas because this measurement reflects the $^{129}$Xe pulmonary gas transfer from alveoli to bloodstream, i.e. RBC:Gas measures $^{129}$Xe transfer across the membrane barrier, but M:Gas only measures increased membrane signal. As highlighted in Figure 1, RBC:Gas is analogous to $K_{CO}$.

Sex was chosen over height for a few reasons:

1. To follow previous work on the age and sex dependence of $^{129}$Xe gas exchange ratios [2, 4].
2. The $^{129}$Xe dosing is chosen based on height, so some adjustment for height is already made in the $^{129}$Xe MRI protocol.
3. There are other physiological differences between males and females which affect gas exchange, such as haemoglobin levels and effects of the menstrual cycle [5].
4. Height was strongly correlated with both sex and VV.

The features for the random forest model were chosen to be the same as the features in the linear regression model. However, with random forest there are no multicollinearity concerns so model performance with the addition of height as a feature was also tested. Including height was found to have no improvement on the model performance (mean

squared error = 1.13 ± 0.24, mean absolute error = 0.995 ± 0.129 without height, mean squared error = 1.13 ± 0.54, mean absolute error = 1.00 ± 0.16 with height). The standard deviation between folds was higher when height was included, suggesting that this feature is unnecessary and is leading to overfitting.

Ultimately, there is no definitive answer about which variables to include in machine learning prediction models such as ours. Factors that have a significant impact on lung function, such as smoking history, were not included in our models because they were not available, but would very likely improve prediction accuracy. With more variables in the prediction models we would expect to see improved fitting of the training data, however with these additional degrees of freedom there is also higher risk of overfitting and the model not performing well on validation data.

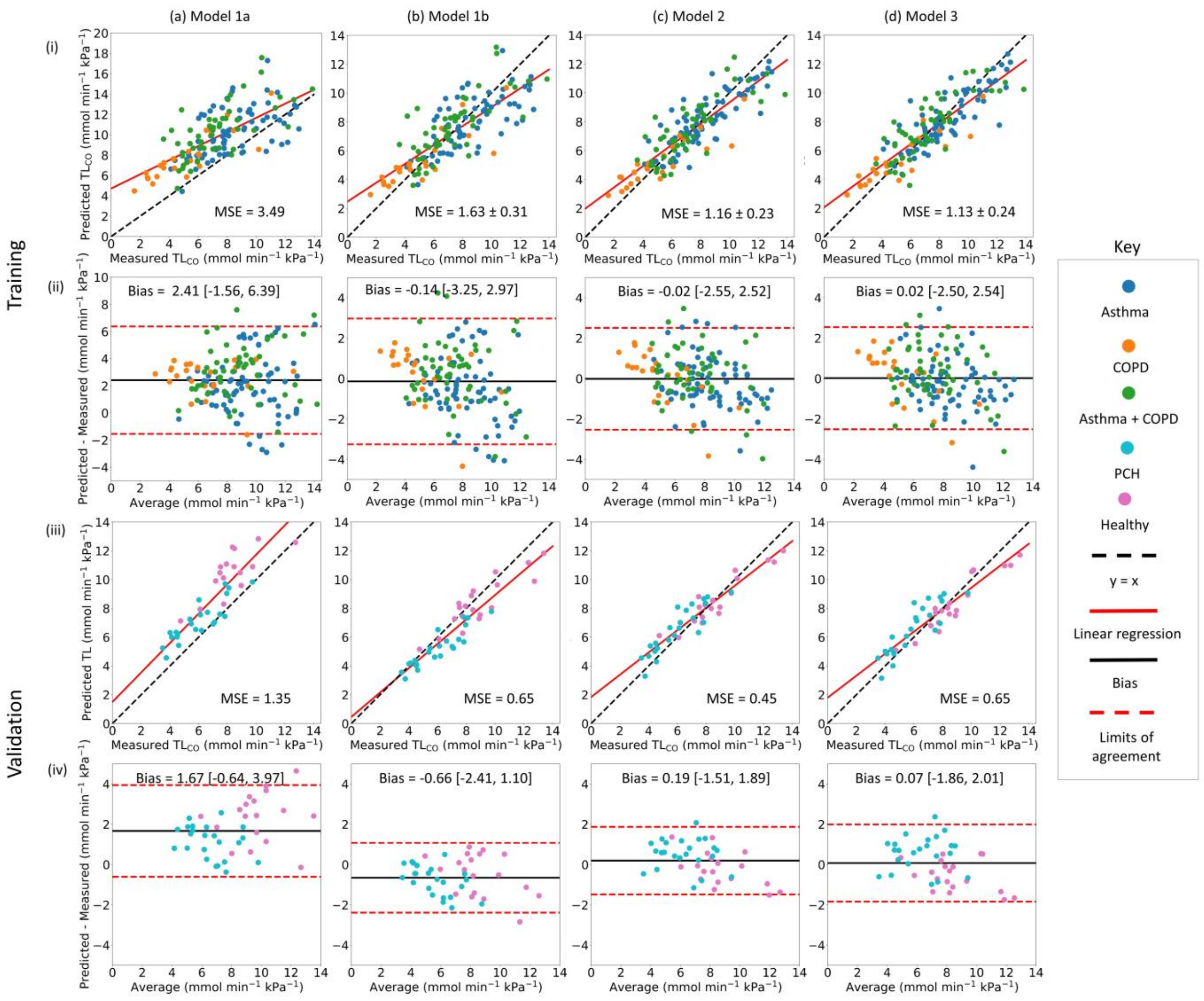


Figure S1: Evaluation of the three prediction models on the training data and validation data: (a) physiology-based model with coefficients from Wang et al [6] and (b) coefficients refitted on our training data, (c) multivariate linear regression and (d) random forest regression, via (i, iii) linear regression and (ii, iv) Bland-Altman plots of the measured and predicted $TL_{CO}$ values. MSE = mean squared error, given as the mean ± standard deviation across the five cross-validation training folds, where applicable. PCH = post-COVID-19 hospitalisation patients.

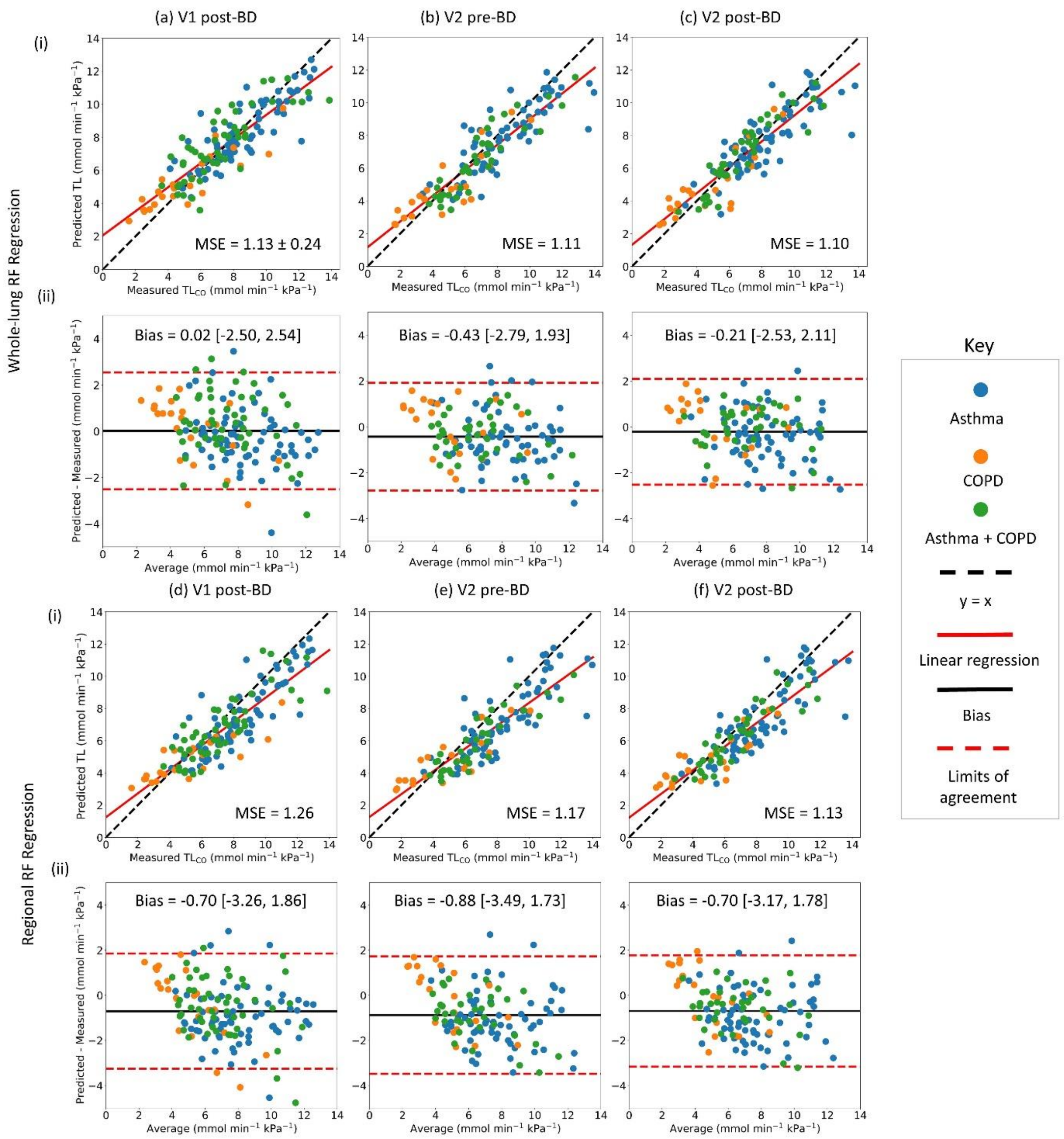


Figure S2: Evaluation of the whole-lung random forest regression model and the regional random forest regression model on data from the Advanced Diagnostic Profiling (ADPro) asthma and chronic obstructive pulmonary disease study for (a) visit 1 (training data), (b) visit 2, pre-bronchodilator and (c) visit 2, post-bronchodilator. The first and third rows show linear regression (i) and the second and fourth rows show Bland-Altman plots (ii) of the measured and predicted $TL_{CO}$ values. MSE = mean squared error.

Table S1: $^{129}Xe$ dose chart for ventilation and gas exchange xenon imaging according to patient height. The rationale for these dose volumes is described in Smith el al [7].

| Height (cm) | Ventilation | Gas Exchange |
|---|---|---|
| > 160 | 500 ml $^{129}Xe$<br>500 ml $N_2$ | 1000 ml $^{129}Xe$<br>0 ml $N_2$ |
| 150 - 160 | 450 ml $^{129}Xe$<br>350 ml $N_2$ | 800 ml $^{129}Xe$<br>0 ml $N_2$ |
| 140 - 150 | 400 ml $^{129}Xe$<br>250 ml $N_2$ | 650 ml $^{129}Xe$<br>0 ml $N_2$ |
| 130 - 140 | 350 ml $^{129}Xe$<br>150 ml $N_2$ | 500 ml $^{129}Xe$<br>0 ml $N_2$ |
| 120 - 130 | 300 ml $^{129}Xe$<br>100 ml $N_2$ | 400 ml $^{129}Xe$<br>0 ml $N_2$ |

Table S2: Random forest regression model parameters.

| | Maximum tree depth | Number of estimators |
|---|---|---|
| $V_A$ | 4 | 500 |
| $K_{CO}$ | 3 | 200 |

*Abbreviations: $V_A$ = alveolar volume, $K_{CO}$ = transfer coefficient*

Table S3: The coefficients of the multivariate linear regression model (Equations 11 and 12), given as the mean ± standard deviation across the five cross-validation training folds.

| | $K_{CO}$ | $V_A$ |
|---|---|---|
| VV | N/A | $b_1 = 0.6378 \pm 0.0281$ |
| $\frac{1}{RBC}$ | $a_1 = -0.0015 \pm 0.0001$ | N/A |
| Age | $a_2 = -0.0018 \pm 0.0004$ | $b_2 = -0.0245 \pm 0.0038$ |
| $Sex_F$ | $a_3 = 0.0740 \pm 0.0203$ | $b_3 = -1.3752 \pm 0.0650$ |
| c | $a_4 = 2.0500 \pm 0.0371$ | $b_4 = 4.75 \pm 0.35$ |

*Abbreviations: $K_{CO}$ = transfer coefficient, $V_A$ = alveolar volume, VV = ventilated volume, RBC = $^{129}$Xe red blood cell signal, $Sex_F$ = 1 for females, 0 for males, c = constant term*

## References


1. Collier GJ, Eaden JA, Hughes PJC, et al., *Dissolved 129 Xe lung MRI with four-echo 3D radial spectroscopic imaging: Quantification of regional gas transfer in idiopathic pulmonary fibrosis.* Magnetic Resonance in Medicine, 2021. **85**(5): p. 2622-2633.
2. Collier GJ, Smith LJ, Saunders LC, et al. *Age, sex, and lung volume dependence of dissolved xenon-129 MRI gas exchange metrics*. Magnetic Resonance in Medicine, 2024. DOI: https://doi.org/10.1002/mrm.30133.
3. Ruppert K, Amzajerdian F, Hamedani H, et al., *Assessment of flip angle-TR equivalence for standardized dissolved-phase imaging of the lung with hyperpolarized 129Xe MRI.* Magnetic Resonance in Medicine, 2019. **81**(3): p. 1784-1794.
4. Mummy D, Zhang S, Bechtel A, et al., *Functional gas exchange measures on (129)Xe MRI and spectroscopy are associated with age, sex, and BMI in healthy subjects.* Frontiers in Medicine (Lausanne), 2024. **11**: p. 1342499.
5. Sansores RH, Abboud RT, Kennell C, et al., *The effect of menstruation on the pulmonary carbon monoxide diffusing capacity.* Am J Respir Crit Care Med, 1995. **152**(1): p. 381-4.
6. Wang Z, Swaminathan A, Bier E, et al., *Using (129)Xenon MR Gas Exchange MRI to Measure the Membrane and Capillary Components of DLCO and KCO.* American Journal of Respiratory and Critical Care Medicine, 2020. **201**.
7. Smith LJ, Collier GJ, Marshall H, et al., *Patterns of regional lung physiology in cystic fibrosis using ventilation magnetic resonance imaging and multiple-breath washout.* European Respiratory Journal, 2018. **52**(5): p. 1800821.
